# Thin film ferroelectric photonic-electronic memory

**DOI:** 10.1038/s41377-024-01555-6

**Published:** 2024-08-23

**Authors:** Gong Zhang, Yue Chen, Zijie Zheng, Rui Shao, Jiuren Zhou, Zuopu Zhou, Leming Jiao, Jishen Zhang, Haibo Wang, Qiwen Kong, Chen Sun, Kai Ni, Jixuan Wu, Jiezhi Chen, Xiao Gong

**Affiliations:** 1 Department of Electrical and Computer Engineering, National University of Singapore, Singapore, 119077 Singapore; 2 Department of Microelectronic Engineering, Rochester Institute of Technology, Rochester, NY 14623 USA; 3 School of Information Science and Engineering, Shandong University, Jinan, 250100 China

**Keywords:** Optical data storage, Photonic devices, Nanophotonics and plasmonics, Silicon photonics

## Abstract

To reduce system complexity and bridge the interface between electronic and photonic circuits, there is a high demand for a non-volatile memory that can be accessed both electrically and optically. However, practical solutions are still lacking when considering the potential for large-scale complementary metal-oxide semiconductor compatible integration. Here, we present an experimental demonstration of a non-volatile photonic-electronic memory based on a 3-dimensional monolithic integrated ferroelectric-silicon ring resonator. We successfully demonstrate programming and erasing the memory using both electrical and optical methods, assisted by optical-to-electrical-to-optical conversion. The memory cell exhibits a high optical extinction ratio of 6.6 dB at a low working voltage of 5 V and an endurance of 4 × 10^4^ cycles. Furthermore, the multi-level storage capability is analyzed in detail, revealing stable performance with a raw bit-error-rate smaller than 5.9 × 10^−2^. This ground-breaking work could be a key technology enabler for future hybrid electronic-photonic systems, targeting a wide range of applications such as photonic interconnect, high-speed data communication, and neuromorphic computing.

## Abstract (web summary)

A non-volatile memory with information stored in the polarization of the ferroelectric material and can be read out via capacitance and resonant wavelength.

## Graphical abstract

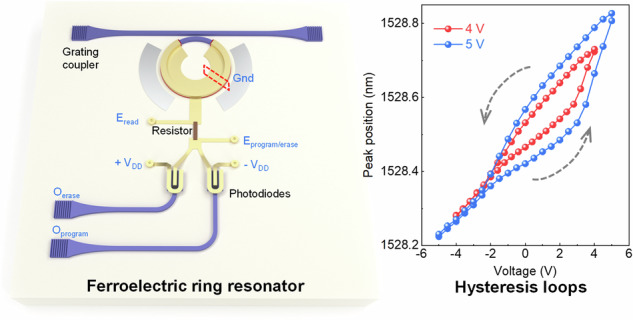

## Introduction

In the era of big data and artificial intelligence, conventional electronic computers based on Von-Neumann architecture struggle to meet the stringent demands of data-intensive and massive parallel tasks^1–3^. To address this challenge, photonic integrated circuits (PICs) hold tremendous promise to offer a potential solution to the increasing need for computing power in the coming decades. Exploring transmission, manipulation, processing, and storage of information using optics and photonics could provide powerful solutions^4–7^, leveraging the inherent properties such as high bandwidth, high power efficiency, low latency, and high parallelism^8–10^. Notable successes have already been achieved in demonstrating PICs for accelerating tasks, such as non-linear programming^11^, signal processing^12–14^, photonic neural networks^15–17^, and quantum computing^18,19^. However, the lack of optically accessible memory poses a significant hurdle in system design, as it necessitates additional electro-optical and opto-electrical conversion processes during computing. This has led to a growing interest in non-volatile photonic-electronic memory as a means to bridge the gap between the electronic and photonic circuits. Currently, control and data processing predominately occur electrically due to the absence of optical memory. This results in additional loss, latency, and power consumption due to moving the data between different components and hinders the realization of optical in-memory computing. Therefore, an ideal memory for hybrid electronic-photonic systems should possess the following features: (a) Non-volatile nature with high endurance, long retention, multi-level storage capabilities, and zero energy consumption to maintain the data storage; (b) The ability to be programmed and erased both electrically and optically; (c) The non-destructive electrical and optical read processes that can occur simultaneously without conflict; (d) Fully compatible with the fabrication process of both electronic and photonic components.

There have been a few existing reports to realize the non-volatile photonic memory^20–22^, with chalcogenide phase change materials (PCM), such as Ge_2_Sb_2_Te_5_ (GST) receiving the most attention in recent years^23–30^. They take advantage of the different absorption coefficients of GST in its amorphous and crystallization states to modify the intensity of the guided light in the waveguide. However, the reported low extinction ratio (ER) could be one of the drawbacks that limit its applications. In addition, the program/erase processes require pulse engineering, increasing the complexity of peripheral circuit design^23^. Moreover, challenges such as resistance drifting^31,32^ arising from structural relaxation, as well as the thermal crosstalk^33,34^ in high-density integration of the PCM memories, are yet to be solved. Another method involves the use of the floating-gate structure, where carrier injection into the floating gate changes the refractive index (RI) of the waveguide in a non-volatile way^35,36^. However, they generally require a high working voltage of a few tens of volts and operate at a low speed of a few Hz. Low program/erase endurance is another issue arising from the accumulation of interface traps in the carrier injection process^37,38^. Another method employs the memristor structure where a conductive bridge is created inside the a-Si cladding layer above the Si waveguide using electrical force, resulting in non-volatile modulation of the waveguide^39–41^. However, this approach faces the challenges of poor retention, low endurance, highly stochastic switching, etc^42^. Importantly, most reported non-volatile photonic memory implementations are not electrically readable and electrically controllable, which further narrows their scope of applications in optoelectronic circuits. Furthermore, all the non-volatile photonic memory demonstrations lack a systematic investigation of the endurance and retention properties of the memory cell, which are the key figures of merit (FOMs) in all memory applications.

Ferroelectricity discovered in the doped-HfO_2_ thin film has garnered significant attention as a technology enabler for emerging ferroelectric (FE) memory^43–46^. Compared with FE materials in complex perovskite systems^47–49^, doped-HfO_2_ shows great promise and better performances for non-volatile device applications due to its full CMOS compatibility, high scalability, advantage of retention characteristics, and the ability to maintain ferroelectric properties down to nanometer-level thickness^50,51^. In this work, we demonstrate a non-volatile multi-level photonic-electronic memory by introducing an Al-doped HfO_2_ (HAO) FE thin film onto a silicon photonic platform. A 3D monolithic integrated structure introduces the non-volatile property directly on the silicon waveguide, without any additional energy to maintain the optical information. The memory is programmable and erasable both electrically and optically at 1550 nm telecom wavelength, assisted by optical-to-electrical-to-optical (OEO) conversion, while the optical read function is completely optical. The electrical read and optical read processes do not conflict and can happen at the same time. Using a ring resonator structure, the memory cell achieves one of the highest ERs of 6.6 dB with a low working voltage of 5 V. The performance of the memory is analysed in detail, demonstrating multi-level storage capability with minimal errors. The device exhibits a decent endurance of 4 × 10^4^ cycles. No noticeable change can be observed during the measured 1000 s retention time, and the fitted retention time is estimated to be more than 10 years, proving its good stability and non-volatility. Moreover, our design ensures that the FE thin film is on top of the waveguide and fabrication condition does not affect other photonic components. The fabrication simplicity, size of the unit cell, and the mature large wafer capability provide great potential for our design to scale up. Based on our current results, our non-volatile memory could pave the way for hybrid electronic-photonic systems, with applications including photonic interconnect, high-speed data communication, and neuromorphic computing.

## Results

### Design of non-volatile photonic-electronic memory cell

**Fig. 1:**
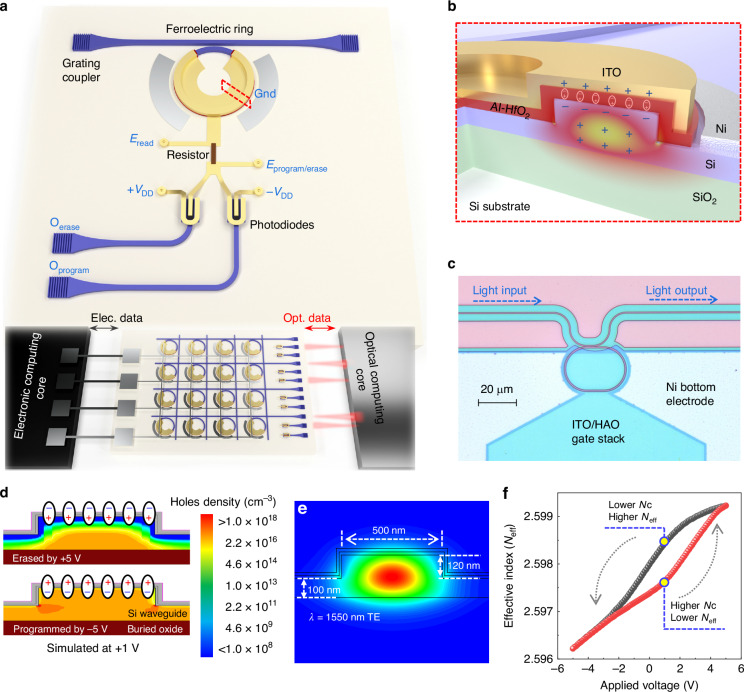
Schematic and simulation results of the non-volatile photonic memory. **a** The 3D schematic of the non-volatile photonic memory cell with both electrical programming/erasing and optical programming/erasing functionalities. Ferroelectric material Al-doped HfO_2_ (HAO) is deposited on top of the silicon waveguide to modulate the carrier concentration in the waveguide, such that the information can be stored in a non-volatile way. Peripheral circuits are designed for mixed-mode operations, for which two photodiodes are used for optical programming/erasing. The optical signal can be read out from the grating coupler of the bus waveguide. Electrical programming/erasing is enabled by the direct electrical bias, then delivered by the metal line to the HAO. Electrical reading relies on the E_read_ port by small signal sensing. This memory can serve as the interface for future hybrid electronic-photonic system to simplify the peripheral circuit design. **b** Cross-sectional zoom-in view on the ring. The Si waveguide is P-type doped to ensure the carrier concentration required by the ferroelectric switching. Light will transmit in the Si waveguide core under the non-volatile modulation of the ferroelectric material HAO. By switching the HAO, carrier concentration inside the waveguide is changed, resulting in a different propagation constant of the light. Therefore, the resonance wavelength of the optical ring resonator will be non-volatilely modified and can be viewed as a stored state. ITO serves as the top electrode to ensure low optical absorption as well as good ferroelectricity. **c** The microscope image of the non-volatile photonic switch. **d** Simulated carrier concentrations at 1 V biasing voltage after being initiated by voltages with opposite polarity. A distinct difference in carrier concentration under these two states is shown. **e** Optical mode profile simulation of the waveguide. Single transverse-electric mode is confirmed. **f** The effective index of the optical mode versus the applied voltages was simulated based on the carrier concentration simulation results. The direction of the hysteresis loop (counter-clockwise) proves the FE memory principle theoretically

The non-volatile photonic-electronic memory cell is based on a ring resonator structure, as illustrated in Fig. 1a, while Fig. 1b provides the zoom-in cross-section of the ring region. The microscope image of the fabricated memory cell is shown in Fig. 1c. Ring resonators are used for their advantage of better tunability, easier design, and fabrication. Light is coupled in and out of the chip by using two partially etched grating couplers. In the ring resonator region shown in Fig. 1b, an FE capacitor is directly fabricated on the Si waveguide using 10 nm-thick HAO as the dielectric layer, offering an effective modulation of the silicon waveguide by the FE polarization. In the capacitor, a 13 nm indium tin oxide (ITO) serves as the top transparent electrode, while the p-type lightly doped Si waveguide with a hole concentration (*N*_h_) around 1 × 10^17^ cm^−3^ serves as the bottom electrode. A larger area of nickel is deposited on the Si bottom electrode to lower the parasitic resistance in the measurement. It should be noted that the fabrication of the FE capacitor is independent of the fabrication of waveguide components, and can be fabricated after all the foundry processes. The anneal of the HAO layer requires only 650 °C temperature, minimizing any effect on other photonic active and passive components.

Upon applying different voltages on top and bottom electrodes, the dipoles inside the HAO film can be switched, after which a remnant polarization (*P*_r_) corresponding to the applied electric field maintains. This *P*_r_ modifies the *N*_h_ in the p-type Si waveguide underneath the HAO, and thereby changes the RI of the waveguide. The free-carrier concentration is changed and held in place by the electrostatic force from the ferroelectric layer, namely the electrostatic doping effect^52^. As shown by the cross-sectional view in Fig. 1b and *N*_h_ simulation results in Fig. 1d, when negative (positive) voltage is applied on the top electrode, the dipoles will be switched to a direction that can attract (repel) the holes, then the *N*_h_ in the Si will be higher (lower). Due to the permanent dipole moment after switching, the change in *N*_h_ is non-volatile and directly related to the *P*_r_. The accumulated (depleted) holes decrease (increase) the RIs and the propagation constant, leading to the blue (red) shift of the resonance peaks. Considering the waveguide dispersion in the submicron silicon waveguide and the resonant phenomenon in the micro-ring, this effect can be quantified by^53^

$$\Delta {\lambda }_{{\rm{res}}}=\frac{\Delta {n}_{{\rm{eff}}}{\rm{\cdot }}{\lambda }_{{\rm{res}}}}{{n}_{{\rm{g}}}}$$

where ∆*n*_eff_ is the effective index change induced by the difference in carrier concentration; *n*_g_ is the group index; *λ*_res_ is the center wavelength of one resonance peak; ∆*λ*_res_ denotes the wavelength shift. This shifted resonance peak position can then be optically read out by either measuring the optical spectrum when the input is a broadband light or the output power when the input is a tuneable laser aligned to a resonance peak. Non-destructive electrical reading is achieved by sensing the capacitance change using a small electric signal before and after the FE switching^54^. Noted that the readings are based on different mechanisms, thus the optical reading and electrical reading can operate independently without any conflict in future electronic-photonic systems as shown in Fig. 1a.

We aim to design a photonic-electronic memory that is capable of mixed-mode operations, where optical/electrical programming/erasing are all feasible. As shown in Fig. 1a, electrical programming/erasing biases can be directly applied from the *E*_program/erase_ port to control the FE switching. Optical programming/erasing will rely on two photodiodes (PDs) to convert the optical power to electrical bias. These two PDs are constantly reverse-biased. When optical power is presented on the PDs, PDs serve as current sources, which can deliver the applied reverse-bias voltages to the FE capacitor assisted by a pull-up resistor. In this way, optical power can be used for programming/erasing. Upon programming/erasing, the results will be read out from the grating coupler optically, or from the *E*_read_ port electrically.

Based on the device design, the simulations have been conducted on the ring region accordingly to confirm the working principle, as shown in Fig. 1d–f for *N*_h_ distribution, optical mode profile, and *n*_eff_ simulation, respectively. The FE capacitor on the Si waveguide is erased/programmed using voltages with different polarities and read at 1 V. Note that the 1 V reading voltage provides a larger memory window but not necessary. The device shows decent memory window at 0 V. The non-volatility does not depends on the reading voltage. Pronounced differences in *N*_h_ (> 8 orders difference at +1 V reading voltage) are found in the Sentaurus TCAD simulation in Fig. 1d, which is expected due to the large and opposite *P*_r_ after programming and erasing. The waveguide is designed to have an etching depth of 120 nm and a width of 500 nm. Single-mode operation is confirmed by the Lumerical MODE simulation, shown in Fig. 1e. Due to the large *N*_h_ difference, the simulated optical effective index response in Fig. 1f shows a counter-clockwise hysteresis loop representing the effect of ferroelectric-induced modulation of *N*_h_ on optical phase, which can be further converted to resonant peak shift.

### Non-volatile photonic-electronic memory cell performance

**Fig. 2:**
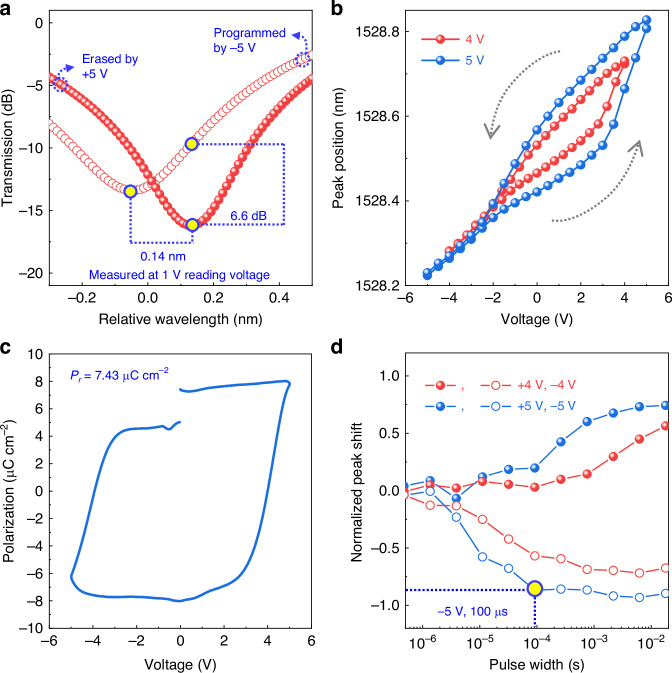
Measurement results of the non-volatile photonic memory. **a** Measured transmission spectra of the ring resonator at different voltages. The device was erased by a 5 V pulse first and then programmed by a −5 V pulse. A high extinction ratio of 6.6 dB and a peak difference of 0.14 nm were obtained at 1 V reading voltage. **b** Measured resonance peak position versus the applied voltages. The counter-clockwise hysteresis loops are caused by ferroelectricity. **c** *P*-*V* loops measured by PUND method of the device with 10 nm HAO, featuring a remnant polarization of 7.43 μC cm^−2^. **d** Measured resonance peak shift versus the input pulse width with pulse amplitude of 4 or 5 V. 90% of the maximum peak shift can be achieved by a −5 V, 100 μs pulse, featuring a 10 kHz writing speed of the memory cell

The devices were fabricated after the device design and the simulation confirmation. Detailed fabrication processes, SEM/TEM characterizations, and the electrical characterizations of the FE film can be found in the Method section as well as the supplementary material Fig. S1 and S2. When measuring the devices, the operational speed is limited by the carrier generation/recombination time of Si for the FE switching process^46^. Therefore, all the measurements were carried out with a visible laser irradiating on the ring region to boost the electron generation/recombination speed. The power spectra (Fig. 2a) of the optical ring resonator were measured under a reading voltage of 1 V after programming and erasing using −5 V and 5 V, respectively. An excellent ER of 6.6 dB is shown, which is one of the highest ER among the reported works considering the low voltage used. For practical applications, photodetectors will be used to read the stored state. In this case, a high ER is preferred as it can lower the bit-error-rate (BER) of the stored information. A maximum peak difference of 0.14 nm is also featured in this test. Quality factors of the resonance peaks are different in these two states, indicating that the carrier concentrations are varying in different states. Detailed studies of the quality factor are shown in supplementary material Fig. S3. In addition to the spectra, we extracted the resonance peak positions and plotted them in Fig. 2b, in which clear counter-clockwise hysteresis loops can be observed for both 4 V and 5 V sweeping voltages. The hysteresis direction is critical here because it can validate the working principle. When the biasing voltage is sweeping from −5 V to 5 V, the FE layer HAO is first programmed by −5 V, resulting in hole accumulation in the waveguide. The effect of hole accumulation is retained by the *P*_r_ of the HAO layer, so the resonance peaks in the forward (−5 V to 5 V) sweeping is blue shifted compared with the resonance peaks in the backward (5 V to −5 V) sweeping. The reading voltage is selected as 1 V to maximize the output difference at different states. The transmission hysteresis loop is also calculated and provided in supplementary material S4. The electrical memory properties are also confirmed from the polarization-voltage relationship measured using the positive-up-negative-down (PUND) method, as shown in Fig. 2c. The experimentally measured hysteresis loop is direct evidence for the proper functioning of our non-volatile photonic-electronic memory.

As an integral part of all memory applications, the speed performance of programming, reading, and erasing operations is important. Here, to determine the high-speed performance of the memory cell, we examined the operation speed by using electrical pulses with the same amplitude (4 V or 5 V) but varying pulse width (ranging from 2 ns to 2 ms). The amount of resonance peak shift is used to assess the switching performance under each applied electrical pulse, as depicted in Fig. 2d. The result shows that 90% of the maximum resonance peak shift can be achieved by a −5 V, 100 μs electrical pulse. Higher voltage generally offers a higher operating speed due to a larger electric field across the FE layer. Taking the p-type substrate into account, the switching of the HAO FE layer is easier when applying negative voltages than positive voltages because more holes in the Si waveguide are available to respond to the FE switching (see electrical characterizations of FE switching speed in the supplementary material Fig. S2). For our memory cell, negative voltages are used for the programming process since the speed for information storage is more important.

### Demonstration of multi-mode and multi-level storage functions

**Fig. 3:**
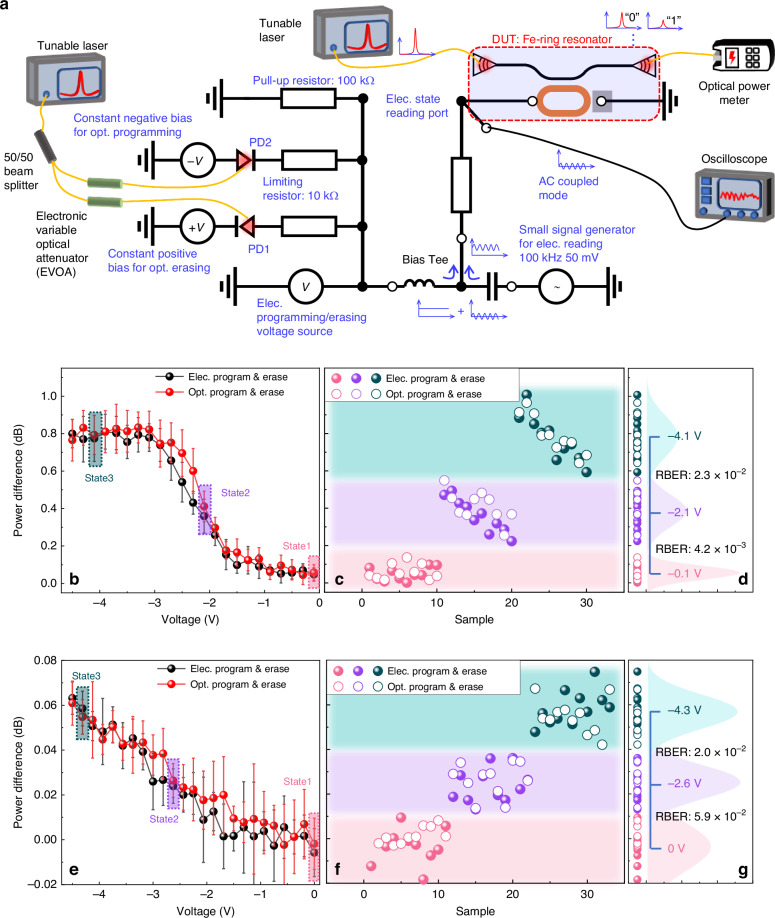
Measurement setups and results for mixed-mode erasing/programming operation. **a** The measurement setups for mixed-mode operation. Optical erasing/programming is realized by two constantly reverse-biased PD1/PD2, respectively. Two EVOAs are used to adjust the optical power on the PDs and the photocurrent from the PDs, and finally, it controls the DC bias being delivered to the bias tee. Electrical erasing/programming was carried out by a single voltage source. Optical reading was then enabled by an optical power meter, while an oscilloscope provided the electrical reading function. **b** The optical power differences are read out at 1 V reading voltage after each erasing (4.5 V)/programming (−0.1 V ~ −4.5 V) operation in both optical/electrical ways. Due to the losses and noises in both the electrical circuit and the optical circuit, small deviations are shown between these two curves. Taking the losses and noises in both electrical/optical ways into consideration, three states can be clearly distinguished, which are state1 (−0.1 V), state2 (−2.1 V), and state3 (−4.1 V), corresponding to non-switched, partially-switched, and fully-switched FE state, respectively. The error bars are defined by the standard deviation of 10 data points. **c** Data points are shown in this figure for electrical operation (solid circles) and optical operation (hollow circles). **d** To verify the practicality of the multi-level operation, RBERs are calculated between each consecutive state. Here, the highest RBER is 2.3 × 10^−2^ between state1 and state2, which can be lowered to less than 10^−12^ by LDPC coding. **e** The electrical reading function of this readout circuit. Power differences are read out from an oscilloscope by sensing the magnitude of the small signal at 100 kHz. Considering the small deviations between electrical operation and optical operation, similar three states can be used for data storage, corresponding to non-switched and fully-switched state, respectively. The error bars are defined by the standard deviation of 10 data points. **f** Data points for these two states are shown for electrical operation (solid circles) and optical operation (hollow circles). **g** A maximum RBER of 5.9 × 10^−2^ is calculated between these two states, showing great reliability of the electrical readout function of the designed circuits

Taking advantage of the FE material, where the external voltage bias can control the induced *P*_r_ with partial switching capability, our non-volatile photonic-electronic memory is able to realize multi-level storage in a simple manner. By applying different programming voltages, the maximum achievable peak wavelength differences and ERs can be controlled by the induced *P*_r_. Multi-level storage, as well as the mixed-mode operation, are proved to be feasible here, with details shown in Fig. 3. The measurement circuit design is shown in Fig. 3a, where optical/electrical programming/erasing/reading functions are equipped. To induce the FE switching and programming/erasing the memory cell, it is crucial to create enough DC bias on the FE capacitor. Here in the circuit, a bias tee is used to deliver the DC voltage from the voltage input (DC port of bias tee) to the device (RF + DC port of bias tee). The DC voltage for programming/erasing can be generated either optically or electrically: (a) Optical programming/erasing is realized by two constant reverse-biased PDs, while two electrically controlled EVOAs are used to tune the optical power intensity on these two PDs. Noted that fiber-coupled PDs on separated chips are used in the proof of principle experiment. The output photocurrents from the PDs flow through a pull-up resistor, which can create DC voltages at the DC port of the bias tee. The constant positive/negative DC bias being delivered to the input of the bias tee is therefore related to the optical power on the PDs. (b) Electrical programming/erasing is realized by a voltage source that directly produces the DC voltage into the bias tee, while in the meantime, both PDs are kept in the off state by turning off the optical power using EVOA. After optical/electrical erasing/programming, our memory can be read out both optically and electrically at the same time: (a) Optical readout is conducted by having a tunable laser input aligned with a resonant peak and measuring the output power using an optical power meter, where different storage states result in different optical power outputs. (b) Electrical readout relies on sensing the capacitance using a small signal input at the RF port of the bias tee, which is a non-destructive measurement different from the PUND method. The small signal is generated by a signal generator with a frequency of 100 kHz and a magnitude of 50 mV. The impedance of the ITO/FE/Si capacitor is changed along with the FE switching and can then be read out by the magnitude of the small signal. These working principles of optical/electrical programming/erasing/reading guide the following testing. Note that a small AC signal introduces a negligible difference in the optical reading considering an averaged power reading time of more than 10 μs. Thus, the optical reading and electrical reading can be simultaneously realized.

Multi-level storage was first demonstrated by optical reading after electrically/optically programming/erasing, as shown in Fig. 3b. Starting at 0 V, the memory cell was erased by a + 4.5 V pulse before being programmed by negative voltages ranging from −0.1 V to −4.5 V, and read under a reading voltage of 1 V. The length of the pulse is long enough to ensure the switching of the FE layer between each state. These voltage pulses for programming/erasing are generated either electrically or optically, without the requirement of prior knowledge for the driving source. Considering the noise and drift of the system, three states are available for this device using different programming voltages of −0.1 V, −2.1 V, and −4.1 V, which correspond to non-switched, partially switched, and fully switched FE states, respectively. Raw data points repeating 10 times for these three states are shown in Fig. 3c. The raw bit-error-rate (RBER)^55^ between each consecutive state, which is not larger than 2.3 × 10^−2^, as shown in Fig. 3d.

The memory cell was erased by +4.5 V and then programmed by negative voltages ranging from −0.1 V to −4.5 V. The readout is measured from frequency domain output at 100 kHz in Fig. 3e, showing three clearly distinguishable states with a maximum RBER of 5.9 × 10^−2^ between the states, as illustrated in Fig. 3f and g. Apart from this simple circuit for electrical reading, other methods have been reported on the FE capacitor reading in either a destructive way^56^ or a non-destructive way^54^ with better accuracy.

These RBERs all meet the requirements for utilizing low-density parity check (LDPC) error correction coding and can be smoothly lowered to less than 10^−12^ by LDPC coding^57,58^. Besides the multi-level storage and mixed-mode operation, the simplicity of our multi-level storage realization, in which no pulse engineering is required, is another significant improvement over other implementations of non-volatile photonic-electronic memory. The memory driving system design can therefore benefit from the simplicity of the driving strategy of our memory cell.

**Fig. 4:**
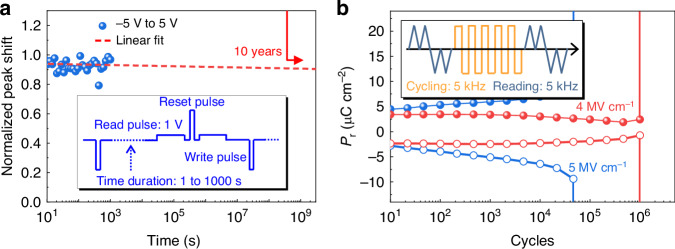
Reliability testing results of the non-volatile photonic memory. **a** Measured peak shift at 1 V biasing voltage versus retention time at room temperature. Write and reset voltages are −5 V and 5 V, respectively. Retention time ranges from 1 s to 1000 s. No obvious degradation was observed. **b** The endurance measurements of the HAO thin film give a minimum endurance of 4 × 10^4^ cycles at 5 V working voltage and 1 × 10^6^ cycles at 4 V. A low cycling frequency of 5 kHz was used to ensure an effective stressing to the HAO thin film. Solid and hollow circles are for positive voltages and negative voltages, respectively

To verify the non-volatile property as well as the reliability of our memory cell, retention, and endurance tests are conducted, as depicted in Fig. 4. In the inset of Fig. 4a, the memory cell was erased and programmed by ±5 V pulses and read under a biasing voltage of 1 V after a time duration ranging from 1 s to 1000 s at room temperature. No obvious degradation was observed within the 1000 s measurement time, and the fitting shows more than 10 years of estimated retention. The retention tests here show the non-volatility of our memory cells. The endurance tests were conducted on a dummy FE capacitor which excludes all the parasitic capacitance in our device. As shown in Fig. 4b, a square pulse train with a cycling frequency of 5 kHz was used to ensure effective electrical stressing and switching of the FE capacitor. The electrical field amplitudes were set to be 4 and 5 MV cm^−1^, corresponding to the operating voltages of our memory cell. The endurance of our HAO thin film increases with a decreasing E-field. A minimum endurance of 4 × 10^4^ cycles at 5 V operation voltage and 1 × 10^6^ cycles at 4 V is obtained. This can be improved by interface engineering between Si and HAO layers^59–61^.

## Discussion

**Fig. 5:**
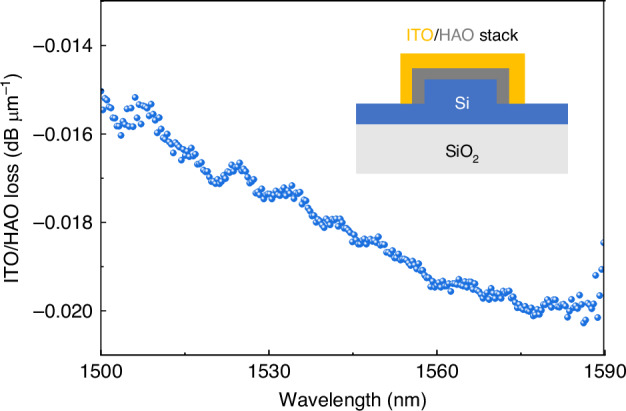
Loss test of the ITO/HAO gate stack on Si waveguide. 13 nm ITO and 10 nm HAO were deposited on the Si waveguide, which is the same gate stack as the memory cell. The loss from the Si waveguide itself was excluded. In this work, ITO/HAO covers about 70 μm waveguide in the ring resonator region, resulting in an acceptable optical loss of about 1.26 dB

In our memory cell, the BER for electrical reading can be improved by implement a capacitance readout circuit^54,56^. The BER for optical reading is mainly limited by the ERs and Q-factors of the optical ring resonator. The extinction ratio can be improved by adding a tunable coupler in the ring to match the critical coupling condition, while the Q-factors are related to the loss of the waveguide. Waveguide loss is strongly related to the lithography and plasma etching step, which have not been fully optimized in this work. Much better results can be expected if the process in commercialized foundries is utilized with waveguide loss of less than 1 dB cm^−1^. Understanding the additional loss caused by the ITO/HAO gate stack is more important here. Measurements were conducted on the Si rib waveguide with ITO/HAO stack on it, as depicted in the inset of Fig. 5. The additional loss of ITO/HAO stack per transmission length was tested and calculated by subtracting the loss of the bare Si rib waveguide. In Fig. 5, additional loss for ITO/HAO is measured to be 0.018 dB μm^−1^, which shows the transparency of our ITO thin film at telecommunication wavelength. In our device, the ITO/HAO covers about 70 μm length on the ring region, corresponding to an acceptable optical loss of around 1.26 dB for this application. A trade-off has to be made here between the conductivity and the transparency of the ITO thin film. A better conductivity results in a more severe photon absorption in the ITO film, degrading the Q-factors of the optical ring resonator and ERs of the memory cell. By lowering the carrier concentration of the ITO thin film in the deposition process, the transparency enhances, but higher parasitic resistance of the ITO/HAO/Si MIS capacitor would degrade its high-speed performance. A possible solution is to reduce the thickness of ITO thin film while keeping its conductivity using the post-annealing process after ITO deposition^62^. Other transparent electrodes can also be used, such as ZnO^63^ and CdO^64^.

In the current proof of principle stage, the full single-chip integration of resistors, PDs, and bias tee, is not achieved. The discrete components might bring in noise and loss in the measurement process. On-chip components are necessary to improve overall performance. Actually, on-chip waveguide-coupled PDs^65^, on-chip resistors, and on-chip bias tee^66^, have already been reported. The integration with other photonic and electronic components for the future hybrid electronic-photonic system is straightforward as the structure is designed to be fully compatible with photonic foundry processes. The maximum temperature during the FE layer fabrication, approaching 650 degrees Celsius, is unlikely to affect the doping profile, the material properties and device performance. One of the potential issues for the full integration is the heat crosstalk. The FE photonic memory cell has negligible heat generation due to its capacitor-like nature. The supporting electronic components can be placed at a distance to isolate the heat transmission. To further improve the thermal stability, active wavelength locking techniques can be introduced as an alternative to the Peltier cooler.

The performance is also not superior to mature technologies, but mainly due to the material contamination and lack of process optimization. The switching speed is mainly limited by the insufficient carrier concentration of the silicon waveguide layer, which can be improved by optimizing the doping profile in the waveguide^54^. Besides, reducing the parasitic capacitance and increasing ITO conductivity will also help increase the speed by lowering the RC delay. The main limitation for the endurance is the defect density of the Si-HAO interface, which can be improved by interface engineering and optimizing the fabrication process^59–61^. Despite not demonstrating an array, our monolithic integration process provides high scalability. The fabrication complexity remains constant irrespective of the device number. Using photonic foundry processes, the unit cell size can also scale down to 5 µm, allowing a 1000 × 1000 array within a typical chip size of 20 × 20 mm.

In conclusion, we have reported here a non-volatile programmable photonic-electronic memory cell with multi-level storage and mixed-mode operation functionalities, enabled by the employment of an Al-doped HfO_2_ ferroelectric layer with low operating voltage and a transparent ITO electrode with low optical absorption on top of the silicon waveguide. In our demonstration, the information is stored optically/electrically induced by the remnant polarization of HAO and can be read out optically by the optical power meter. We achieve one of the highest ERs of 6.6 dB at a 5 V operation voltage, with an endurance of 4 × 10^4^ cycles and an estimated retention of more than 10 years. A comparison with state-of-the-art nonvolatile photonic memories is shown in Table 1. This work shows the considerable potential of ferroelectric photonic devices for future hybrid electronic-photonic platforms, such as photonic interconnect, optical neuromorphic computing, data center, and optical communication applications, where non-volatile photonics are indispensable.

**Table 1:** Performance comparison among state-of-the-art nonvolatile photonic memory technologies

Ref.	Method/ material	Mixed mode	Multi-level	Endurance (cycles)	Retention	Extinction ratio (dB)	Writing speed	Writing voltage	Writing power
Li [23]	PCM: GST	OR OW	✓	\	\	1.55	250 ns	\	680 pJ
Zhou [24]	PCM: GST	OR EW	✓	100	\	4.13	50 ns	7 V	7 nJ
Farmakidis [25]	Plasmonic PCM: GST	✓	✓	\	\	0.0305	20 ns	0.35 V	16 pJ
Delaney [26]	PCM: Sb_2_Se_3_	OR OW	×	\	\	7	400 ns	\	14 nJ
Zhang [28]	PCM: GSST	OR OW EW	×	\	\	42	1 μs	24 V	5.5 µJ
Chen [29]	PCM: SST	OR ER EW	✓	>100	1 h	0.088	2 ns	2.45 V	\
Meng [30]	PCM: GSSe	OR EW	✓	>5 × 10^5^	\	12	500 ms	10 V	400 nJ
Song [35]	Floating Gate: Poly-Si	OR EW	✓	\	\	12.7	600 ms	20 V	20 pJ
Grajower [36]	Floating Gate: SiN	OR EW	×	\	\	0.4	\	110 V	\
Tossoun [39]	Memristor: InP	OR ER EW	✓	>1 × 10^3^	12 h	12.7	300 ps	5 V	0.36 pJ
Emboras [40]	Plasmonic Memristor: Ag	OR EW	×	\	\	0.04	10 ms	9 V	750 pJ
This work	Ferroelectric-SOI: HAO	✓	✓	>1 × 10^6^ @ 4 V	1000 s (measure) 10 yrs. (fit)	6.6	100 μs	5 V	5.05 nJ

*OR* optical read, *OW* optical write, *ER* electrical read, *EW* electrical write

## Methods

### Device fabrication process flow

The ring resonator and the grating coupler were patterned using the Raith EBPG-5200 electron beam lithography system and etched by Oxford PlasmaPro 100 Cobra ICP RIE etching system using CHF_3_/O_2_/Ar on an SOI wafer. 250 nm SiO_2_ was then deposited by Oxford PlasmaPro 100 PECVD as the cladding layer, followed by the opening of an operational window directly on the ring resonator region using dry etching. After that, 10 nm HAO was deposited using Picosun ALD at 300 °C. The doping concentration of aluminum is 1:30 controlled by the cycle ratio of Al and Hf. Tungsten was deposited by Sputtering System - AJA UHV as the top electrode. Rapid thermal annealing was performed by the RTP system from Annealsys. After that, Tungsten top electrode was replaced by ITO top electrode deposited using RF-sputtering by Sputtering System - AJA UHV. Finally, the bottom electrode Ni was deposited by the ebeam evaporation system E-Beam Evaporator - AJA UHV. Detail flow charts and TEM figures can be found in supplementary materials Fig. S1.

### Measurement setup for the mixed mode operations

For the optical programming/erasing function, the output from a C-band EDFA (Fiberprime) is split into two paths and followed by 2 EVOAs (Thorlabs) to control the optical power. InGaAs fiber pigtailed PDs (Beijing Lightsensing) are used to convert the optical power to DC voltages and then applied to the FE memory cell through a bias tee (Marki Microwave). For electrical programming/erasing function, the DC voltages are generated by a source measurement unit (Keysight U2722A). For the optical reading function, the input is a tunable laser (Agilent 81949 A), while the output power is measured by an optical power meter (Thorlabs PM100D). For electrical reading, the small signal is generated by an arbitrary waveform generator (Keysight 33500B) and read by using an oscilloscope (LeCroy WavePro). A visible laser (Thorlabs) with 520 nm wavelength and about 5 mW power is used to help the FE switching and a thermoelectric cooler (TEC) is used to reduce the frequency drift. All the optical spectra and peak shifts are measured by an optical spectrum analyser (Yokogawa AQ6370B).

### Supplementary information

Supplementary information for: Thin film ferroelectric photonic-electronic memory

## Data Availability

All of the data that support the findings of this study are available in the main text. Source data are available from the corresponding author on request.
