# Supplementary material for: Thin film ferroelectric photonic-electronic memory
Source: Light Sci Appl. 2024 Aug 23;13:206. doi: 10.1038/s41377-024-01555-6 (PMC11344043; doi:10.1038/s41377-024-01555-6)
Supplement: Supplementary file 1 — Supplementary information for: Thin film ferroelectric photonic-electronic memory [file 41377_2024_1555_MOESM1_ESM.docx]

**Supplementary information for**

**Thin film ferroelectric photonic-electronic memory**

Gong Zhang^†,1^, Yue Chen^†,1^, Zijie Zheng^1^, Rui Shao^1^, Jiuren Zhou^1^, Zuopu Zhou^1^, Leming Jiao^1^, Jishen Zhang^1^, Haibo Wang^1^, Qiwen Kong^1^, Chen Sun^1^, Kai Ni^2^, Jixuan Wu^3^, Jiezhi Chen^3^,

& Xiao Gong^1,^*

^1^Department of Electrical and Computer Engineering, National University of Singapore, 119077, Singapore;

^2^Department of Microelectronic Engineering, Rochester Institute of Technology, NY 14623, USA;

^3^School of Information Science and Engineering, Shandong University, 250100, China;

^†^These authors contributed equally;

*Corresponding author, Email: elegong@nus.edu.sg, Telephone: +65 65167871.

**S1 Device fabrication processes and characterization results**

The detailed fabrication flow is shown in Fig. S1a. The grating couplers and waveguides were patterned in the first step using electron beam lithography and ICP-RIE dry etching. Partial etching created the Si slab at the ring region for the electrical connections. The Fig. S1b and S1c show the SEM figures of the grating coupler and directional coupler after patterning. After that, PECVD SiO2 served as the cladding layer, followed by the operation window patterning, which can protect the grating coupler and the direction coupler while exposing the optical ring resonator region. The FE layer deposition was done using ALD at 300 ℃. The doping concentration of aluminum is 1:30 controlled by the cycle ratio of Al and Hf. W was deposited as the top electrode. RTA was performed to form the FE phase in Al-doped-HfO_2_. After that, W top electrode was replaced by ITO top electrode deposited using RF-sputtering to ensure a low optical absorption from the top electrode. This replacement gate process is demonstrated with flow chart in Fig. S1d. By utilizing the replacement gate process, we make use of the transparency of ITO without sacrificing the ferroelectricity because no further high-temperature annealing process is needed, and the In and oxygen diffusion problems are solved^1,2^. Finally, the bottom electrode Ni was deposited. The TEM view shown in Fig.S1e reveals the crystallinity of the HAO layer with a thickness of 10 nm. The top-view SEM image of the completed device is illustrated in Fig. S1f.

**S2 Electrical characterization of the ITO/HAO/Si ferroelectric capacitor**

The electrical properties of an ITO/HAO/Si FE capacitor are used to verify the fabrication process and material quality. The electrical measurement setup is shown in Fig. S2a. A visible laser is used to increase the generation speed of Si, which in turn helps the switching process. The P-V loops in Fig. S2b illustrate the full switch of the ferroelectric layer with a remnant polarization of 8.11 μC cm^-2^ at a 5 V sweeping voltage range. Partial switching is realized if a lower sweeping voltage (4 V) is used, which gives a smaller remnant polarization of 4.02 μC cm^-2^. C-V curves with clockwise hysteresis loops in Fig. S2c demonstrate clear ferroelectricity of the HAO film for a capacitor with a device area of 100 × 100 um^2^ as the polarization modulates the depletion width of the P- Si layer^3^. The C-V measurement was carried out at a frequency of 100 kHz with illumination. The I-V measurement results are shown in Fig. S2d, where the switching current peaks are clearly observed at both positive voltage and negative voltage.

Switching speed was also investigated by using different pulse widths (Fig. S2e). A negative square pulse of -5 V, 500 μs was sufficient to induce 90% of the maximum *P*_r_. It should be noted that the device responds faster to negative pulses than positive pulses due to holes as the majority carriers in the p-type Si layer. For positive pulses, the required number of electrons for polarization switching is limited by the carrier generation time in the Si layer. Fig. S2f shows the retention characteristic. Devices were found to have retention of more than 10 years at room temperature from the extrapolated lines.

**S3 Optical characterizations of the ring resonator**

During the switching process, the quality factor will vary with the resonance peak position, as shown in Fig. S3. This is because during the switching process, the blue shift of the resonance peak is related to the carrier accumulation, which may increase the free carrier absorption of the waveguide, and finally result in a degraded quality factor.

**S4 Transmission hysteresis loops**

Besides the wavelength hysteresis loop, the transmission hysteresis loop is also calculated and shown in Fig. S4. The wavelength is chosen such that provides the largest extinction ratio. The result shows that the transmission memory window at 1 V bias is 2.9 dB for 4 V writing voltage and 4.0 dB for 5 V writing voltage.

1. Ryu, T.-H., Yoon, S.-J., Na, S.-Y. & Yoon, S.-M. Crystallization annealing effects on ferroelectric properties of Al-Doped HfO_2_ thin film capacitors using indium–tin–oxide electrodes. *Current Applied Physics* **19**, 1383–1390 (2019).

2. Hu, Y. *et al.* Origin of indium diffusion in high-k oxide HfO_2_. *ACS Applied Materials & Interfaces* **8**, 7595–7600 (2016).

3. Zhou, Z. *et al.* A Metal-Insulator-Semiconductor Non-Volatile Programmable Capacitor Based on a HfAlO*ₓ* Ferroelectric Film. *IEEE Electron Device Lett.* **41**, 1837–1840 (2020).

**Fig. S1 Detail fabrication processes. a** The fabrication process flow for the memory cell. **b c** Zoom-in SEM view at the grating coupler and directional coupler region, respectively. **d** Schematics for the replacement gate process to achieve the ITO/HAO/Si stack. **e** HRTEM image of the ITO/HAO/Si layer stack, delineating the 10 nm thick HAO with poly-crystallinity. The FFT in the inset also indicates the poly-crystalline nature of the HAO. **f** The SEM image of the non-volatile photonic switch. Light paths are denoted in pink.

**Fig. S2 Electrical characterization of the ITO/HAO/Si FE capacitor. a** Measurement setup for electrical characterizations. A green laser was used to speed up the generation time in the Si layer so that the device could respond faster to positive pulses. **b** P-V curves for the MIS capacitor at different sweeping voltages, indicating *P*_r_ of 8.11 μC cm^-2^ for 5 V**. c** Measured C-V curves of the ITO/HAO/Si capacitor. The clockwise hysteresis loops are induced by ferroelectricity. **d** Measured I-V curves for the capacitor at different voltages. **e** Switching speed characterization of the capacitor using electrical pulses with various pulse widths. A -5 V, 500 μs electric pulse could give 90% of the maximum *P*_r_. **f** Retention tests using the FE analyzer. A minimum retention time of 10 years is obtained by retention tests at ±5V switching voltages under room temperature.

**Fig. S3 Quality factors during the sweeping process.** Calculated quality factor versus the resonance peaks position. The red-shifted peaks have higher Q-factors.

**Fig. S4 Transmission hysteresis loops.** The transmission hysteresis loops for different sweeping voltages from -4 V to 4 V (red), and -5 V to 5 V (blue).
